# The Interrelationship between Abscisic Acid and Reactive Oxygen Species Plays a Key Role in Barley Seed Dormancy and Germination

**DOI:** 10.3389/fpls.2017.00275

**Published:** 2017-03-21

**Authors:** Yushi Ishibashi, Nozomi Aoki, Shinsuke Kasa, Masatsugu Sakamoto, Kyohei Kai, Reisa Tomokiyo, Gaku Watabe, Takashi Yuasa, Mari Iwaya-Inoue

**Affiliations:** Crop Science Laboratory, Faculty of Agriculture, Kyushu UniversityFukuoka, Japan

**Keywords:** abscisic acid, barley, catalase, embryo, reactive oxygen species, seed dormancy

## Abstract

Seed dormancy is one of the adaptive responses in the plant life cycle and an important agronomic trait. Reactive oxygen species (ROS) release seed dormancy and promote seed germination in several cereal crops; however, the key regulatory mechanism of ROS-mediated seed dormancy and germination remains controversial. Here, we focused on the relationship between hydrogen peroxide (a ROS) and abscisic acid (ABA) in dormant and non-dormant barley seeds. The hydrogen peroxide (H_2_O_2_) level produced in barley seed embryos after imbibition was higher in non-dormant seeds than in dormant seeds. H_2_O_2_ regulated the ABA content in the embryos through ABA-8′-hydroxylase, an ABA catabolic enzyme. Moreover, compared with non-dormant seeds, in dormant seeds the activity of NADPH oxidase, which produces ROS, was lower, whereas the activity of catalase, which is a H_2_O_2_ scavenging enzyme, was higher, as was the expression of *HvCAT2*. Furthermore, precocious germination of isolated immature embryos was suppressed by the transient introduction of HvCAT2 driven by the maize (*Zea mays*) ubiquitin promoter. *HvCAT2* expression was regulated through an ABA-responsive transcription factor (HvABI5) induced by ABA. These results suggest that the changing of balance between ABA and ROS is active in barley seed embryos after imbibition and regulates barley seed dormancy and germination.

## Introduction

Seed dormancy and germination are crucial stages in a plant’s life. In wild plant species, seed dormancy plays a key role in ensuring survival by blocking germination until conditions become favorable for the later stages of germination and growth of that species (Bewley, 1997). Unlike many wild plant species, cultivated crops such as barley (*Hordeum vulgare* L.) and wheat (*Triticum aestivum* L.) display weak grain dormancy at maturity due to selective breeding against dormancy for uniform and vigorous germination on industrial and agricultural fields. For malting barley, long dormancy increases costs and potential damage resulting from grain storage (Carn, 1980). In addition, cool, moist conditions in the field can disrupt seed dormancy and cause PHS, resulting in serious losses of grain yield and quality (Gubler et al., 2005; Rodríguez et al., 2015). Therefore, research aimed at understanding environmental and genetic controls of dormancy will assist in the development of new strategies for industrial utilization and the elimination of PHS worldwide in domesticated crops.

Plant hormones such as GAs and ABA play key roles in seed dormancy and germination. In particular, ABA is considered to be the key molecule in dormancy induction and maintenance (Finkelstein et al., 2008; Rodríguez et al., 2015; Shu et al., 2016). During seed development, embryonic ABA is required to impose lasting dormancy (Nambara and Marion-Poll, 2005), and *de novo* ABA synthesis in the embryo during imbibition ensures maintenance of dormancy (Kucera et al., 2005). Dormancy release by after-ripening is mediated by a decrease in ABA content in imbibed wheat and barley grains as a result of the coordinated promotion of ABA catabolism and repression of ABA biosynthesis genes (Millar et al., 2006; Jacobsen et al., 2013). In several species, the transcriptional regulation of nine-*cis*-epoxycarotenoid dioxygenase (*NCED*) and ABA-8′-hydroxylase (*ABA8*′*OH*) genes are considered key steps in this control mechanism (Nambara et al., 2010). In barley, *HvNCED1* is particularly important in the regulation of primary dormancy in blue light (Gubler et al., 2008) or at high temperatures (Leymarie et al., 2008), whereas *HvNCED2* expression has been implicated in the induction of primary dormancy (Chono et al., 2006) and the maintenance of secondary dormancy (Leymarie et al., 2008). The *HvABA8*′*OH1* gene plays a major role in that ABA catabolism that is required to alleviate barley grain dormancy (Chono et al., 2006; Millar et al., 2006; Gubler et al., 2008). ABA functions are regulated, in part, by crosstalk with other hormones such as GA and their associated signaling networks. Application of GA, an antagonist of ABA, can break dormancy in cereals (Jacobsen et al., 2002; Tuttle et al., 2015).

Recently, it was reported that several signal molecules such as nitric oxide (NO) and ROS also regulate seed dormancy and germination (Ma et al., 2016). In barley, ROS break seed dormancy and promote germination (Fontaine et al., 1994; Ishibashi et al., 2010; Bahin et al., 2011). The relationships between ROS, seed dormancy, and germination have been described for many plant species, including *Zinnia elegans*, and sunflower (Ogawa and Iwabuchi, 2001; Oracz et al., 2007). Hydrogen peroxide (H_2_O_2_), which is a ROS, is regarded as a signaling hub for the regulation of seed dormancy and germination; the precise regulation of H_2_O_2_ accumulation by the cell antioxidant machinery is essential to achieve a balance between oxidative signaling that promotes germination and oxidative damage that prevents or delays germination (Wojtyla et al., 2016). These findings were clearly summarized and presented by Bailly et al. (2008) as the principle of the “oxidative window” for germination. According to this hypothesis, both lower and higher levels of ROS impair seed germination, which is only possible within a defined range of concentrations.

Recent evidence shows that the selective oxidation of proteins and mRNAs can act as a positive regulator of seed germination (Job et al., 2005; Oracz et al., 2007; Barba-Espín et al., 2010; Bazin et al., 2011). Bazin et al. (2011) showed that approximately 24 stored mRNAs undergo oxidation during sunflower (*Helianthus annuus*) after-ripening. ROS production during germination contributes to reserve mobilization through oxidative modifications of stored proteins; storage organs may then recognize these modifications as signals to mobilize reserves to the rapidly growing axis. Due to the abundance of available seed storage proteins, the oxidized forms of these proteins such as heat shock proteins and elongation factors can also be considered as actor of ROS signaling in seed germination (Job et al., 2005; Barba-Espín et al., 2010). Oracz et al. (2007) proposed a mechanism for seed dormancy release that involves a change in proteome oxidation resulting from the accumulation of ROS during the after-ripening phase.

The breaking of dormancy by ROS has also been reported in relation to plant hormone signaling in several seeds (El-Maarouf-Bouteau et al., 2013). Studies on phytohormone interactions in germinated seeds have shown that exogenously applied ABA inhibits ROS accumulation in barley (Ishibashi et al., 2012), rice (*Oryza sativa*; Ye et al., 2012), lettuce (Zhang et al., 2014), and sunflower (El-Maarouf-Bouteau et al., 2015). By contrast, the addition of GA enhances the production of ROS, mainly superoxide and H_2_O_2_, found in radish plants (Schopfer et al., 2001) and *Arabidopsis* (Liu et al., 2010; Lariguet et al., 2013). Bahin et al. (2011) suggested that exogenously applied H_2_O_2_ does not influence ABA biosynthesis and signaling but has a pronounced effect on GA signaling, resulting in the modulation of hormonal balance and in subsequent germination initiation. The modulation of phytohormone balance during germination by exogenously applied H_2_O_2_ is also a product of changes in H_2_O_2_ levels in seeds treated with GA and ABA. Exogenous H_2_O_2_ and NADPH oxidase inhibitor increased ABA catabolism by enhancing the expression of *CYP707A* genes, which encode ABA 8′-hydroxylases, and enhanced the expression of genes for GA synthesis in dormant *Arabidopsis* seeds (Liu et al., 2010) and non-dormant barley seeds (Ishibashi et al., 2015). In non-dormant barley seeds, H_2_O_2_ accumulation via superoxide produced by NADPH oxidases promote GA biosynthesis in embryos; the resulting GA induces and activates NADPH oxidases in aleurone cells, and the H_2_O_2_ accumulated by the NADPH oxidases induce α-amylase in these cells (Ishibashi et al., 2015). Therefore, it is likely that ROS is central molecule in the regulation of barley seed dormancy and germination through GA and ABA. However, the role of H_2_O_2_ interactions with phytohormones in the regulation of barley seed dormancy and germination is still open to debate. The mechanism of dormancy breaking by ROS needs to be examined in detail. In this study, we, therefore, focused on the balance between plant hormones and ROS in dormant and non-dormant seeds.

## Materials and Methods

### Plant Material

*Hordeum vulgare* L. ‘Ichibanboshi’ grains, which were grown at Kyushu University, were harvested on June 5, 2010. Experiments were carried out with dormant grains that had been stored at -28°C from harvest until the experiments began in order to maintain their initial dormancy (Lenoir et al., 1983). Non-dormant grains were used as controls (i.e., grains from the same harvest but stored for 6 months at 23°C).

### Germination Test

Five replications of 20 seeds each sterilized with sodium hypochlorite were placed on filter paper in a 9-cm Petri dish, to which 6 mL of a solution of 100 mM hydrogen peroxide, 20 mM sodium ascorbate, and distilled water as control was added. The dishes were then incubated in the dark at 22°C (CRB-41L, HITACHI), and the germinating seeds, defined as seeds whose radical protruded through the seed coat, were counted daily for 5 days.

### Hydrogen Peroxide Content

Hydrogen peroxide (H_2_O_2_) content in embryos isolated from seed after imbibition was measured according to the method of Oracz et al. (2007) by using a peroxidase-based assay with 3-dimethylaminobenzoic acid and 1.3 mM 3-methyl-2-benzothiazolinone hydrazone to measure H_2_O_2_ (O’Kane et al., 1996).

### ABA Content

To measure the ABA content in embryos, we isolated embryos from 20 seeds that had imbibed for 48 h and stored them at -80°C. ABA levels were measured by using a Phytodetek Competitive ELISA kit (Agdia). Each experiment comprised five biological replicates.

### Quantitative Real-Time PCR

Total RNA was extracted from embryos isolated after germination treatment or from embryoless half-seeds by using the SDS/phenol/LiCl method (Chirgwin et al., 1979). cDNA synthesis and amplification were conducted as described by Ishibashi et al. (2015). The amount of each gene transcript was normalized against the amount of mRNA for *HvActin* (Trevaskis et al., 2006) by using the method of Pfaffl (2001). The sequences for the *HvActin* primer came from Ishibashi et al. (2015); the other primer sequences are shown in Supplementary Table S1.

### Barley Transient Expression Assay

The plasmid was transformed by particle bombardment into immature embryos according to the method of Pellegrineschi et al. (2002) and Nakamura et al. (2011). For this assay, *H. vulgare* L. ‘Seijo-17’ immature grains, which were grown at Kyushu University, were harvested, and immature embryos isolated from the immature grain. The immature embryos were bombarded with each plasmid, *Ubi:HvCAT* or *Ubi:GUS*, which was constructed to express each gene under the control of the promoter region and the first intron of the maize ubiquitin (*Ubi-1*) gene (Toki et al., 1992; Christensen and Quail, 1996). After bombardment, the immature embryos were transferred to 1/2MS medium containing 200 mg/L MES and 30 g/L maltose, and were incubated at 25°C for 2.5 days. We checked introduction of each genes using RT-PCR (Supplementary Figure S1).

### Electrophoretic Mobility Shift Assay

The EMSA was performed as described by Wang et al. (2011). Recombinant pGEX-HvABI5 proteins were produced in *Escherichia coli* BL21 (DE3) pLysE. The *E. coli* cells were lysed by sonication, and purified with glutathione–Sepharose 4B beads (GE Healthcare). Double-stranded oligonucleotide spanning the ACGT core motif upstream of *HvCAT2* was prepared and carried out labeling according to the protocol provided with the DIG Kit (Roche Diagnostics). EMSAs were carried out using a DIG Gel Shift kit, 2nd Generation (Roche Diagnostics) according to the manufacturer’s instructions. The primer sequences for constructing the pGEX-HvABI5 vector and the probe sequences for the EMSA are shown in Supplementary Table S1.

### Enzyme Activities

To measure enzyme activities, we isolated embryos from 20 seeds that had imbibed for 48 h and stored them at -80°C. The enzyme activities of APX, GPX, SOD, and CAT were measured as described by Ishibashi et al. (2008). NADPH oxidase activity was assayed according to the method of Van Gestelen et al. (1997) and Sarath et al. (2007). Protein concentration was determined by using the method Bradford (1976). The results are expressed as μmol mg^-1^ protein.

## Results

### Germination Rate and Hydrogen Peroxide Content of Dormant and Non-dormant Seeds

**FIGURE 1:**
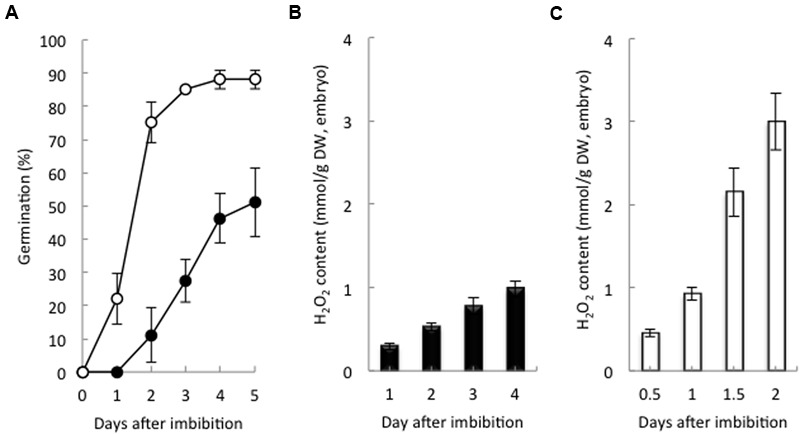
**Germination percentage and hydrogen peroxide content in dormant and non-dormant seeds. (A)** Time course of the percentage of germination of dormant (closed circles) and non-dormant (open circles) seeds. Embryos removed from dormant **(B)** and non-dormant **(C)** seeds after imbibition were used to determine hydrogen peroxide contents (*n* = 5).

Non-dormant seeds had a germination rate of 22% at 1 day after imbibition (DAI), and 85% had germinated at 3 DAI. Dormant seeds had a germination rate of 27 and 50% at 3 and 5 DAI, respectively (**Figure 1A**). At 2 DAI, the H_2_O_2_ content in the non-dormant seeds was higher six times that in the dormant seeds (**Figures 1B,C**). In addition, nine barley cultivars that were at different stages of dormancy had positive correlations with the H_2_O_2_ contents of their embryos after imbibition (Supplementary Figure S2). These data strongly suggest that non-dormant seeds accumulate H_2_O_2_ in the embryo after imbibition, but dormant seeds do not.

### Hydrogen Peroxide and Sodium Ascorbate Regulate Barley Seed Germination through ABA Catabolism

**FIGURE 2:**
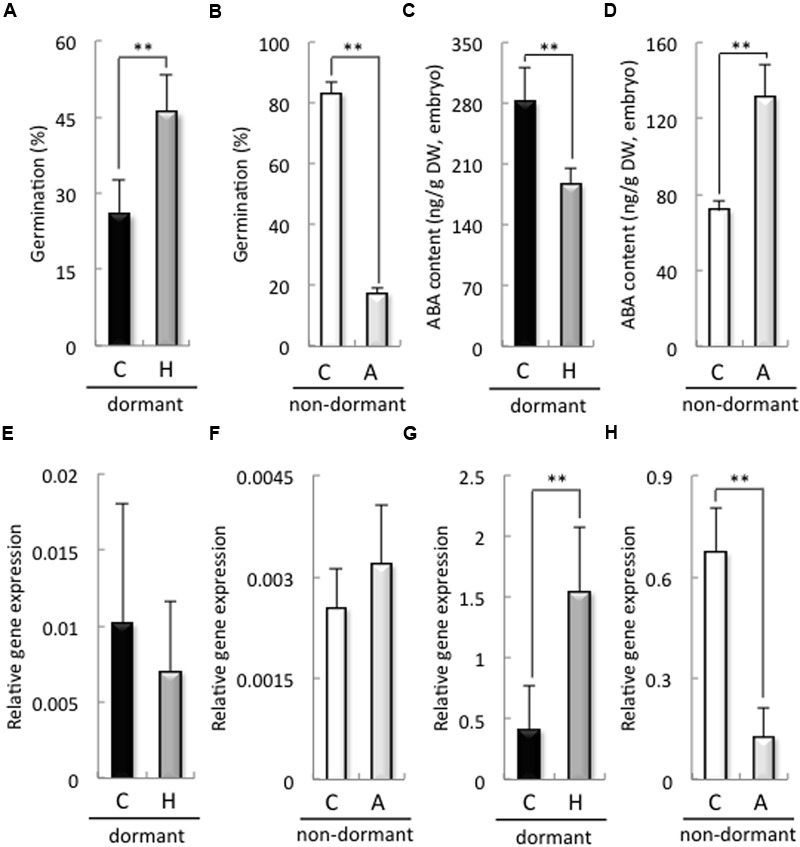
**Germination rate, ABA contents, and expression of ABA metabolism-related genes in dormant and non-dormant seeds.** Embryos removed from seeds after 48 h imbibition were used to determine ABA contents and gene expression. **(A,B)** Germination rate after 48 h imbibition; **(C,D)** ABA contents; **(E,F)** *HvNCED1* expression; **(G,H)** *HvABA8*′*-OH1* expression. **(C)** control (distilled water); **(H)** 100 mM hydrogen peroxide; **(A)** 20 mM sodium ascorbate. (^∗∗^*P* < 0.01, Student’s test, *n* = 5).

The germination rate of dormant seeds treated with H_2_O_2_ was significantly higher than that of the control (**Figure 2A**). In contrast, the germination rate of non-dormant seeds treated with sodium ascorbate was significantly lower than that of the control (**Figure 2B**). In addition, H_2_O_2_ and sodium ascorbate treatments were increased and decreased the H_2_O_2_ contents in the embryos after imbibition, respectively (Supplementary Figure S4). We previously reported that a decrease in ROS, induced by the NADPH oxidase inhibitor DPI, in the embryos of barley seeds suppressed ABA catabolism in those embryos after imbibition (Ishibashi et al., 2015). Therefore, we examined ABA content and biosynthesis, as well as the expression of ABA catabolism-related genes, in the embryos of dormant or non-dormant seeds treated with sodium ascorbate or H_2_O_2_, respectively. In the dormant seeds, the ABA content of the embryos treated with H_2_O_2_ was significantly decreased compared with that in the control (**Figure 2C**). In the non-dormant seeds, the ABA content of the embryos treated with sodium ascorbate was significantly higher than that in the control (**Figure 2D**). The expression of *HvNCED1*, one of the ABA biosynthesis-related genes and a key gene regulating primary dormancy of barley (Millar et al., 2006), in the embryos did not differ significantly regardless of dormancy or treatment (**Figures 2E,F**). However, the expression of *ABA8*′*-OH1*, one of the ABA catabolism-related genes and a key gene regulating primary dormancy of barley (Millar et al., 2006), in the embryo, was suppressed by sodium ascorbate in the non-dormant seeds and increased by H_2_O_2_ in the dormant seeds (**Figures 2G,H**). These results indicate that ROS regulate ABA catabolism in the embryos of non-dormant and dormant seeds by regulating HvABA8′-OH1.

### Activities of Antioxidant Enzymes and NADPH Oxidase in the Embryos of Non-dormant and Dormant Seeds

**FIGURE 3:**
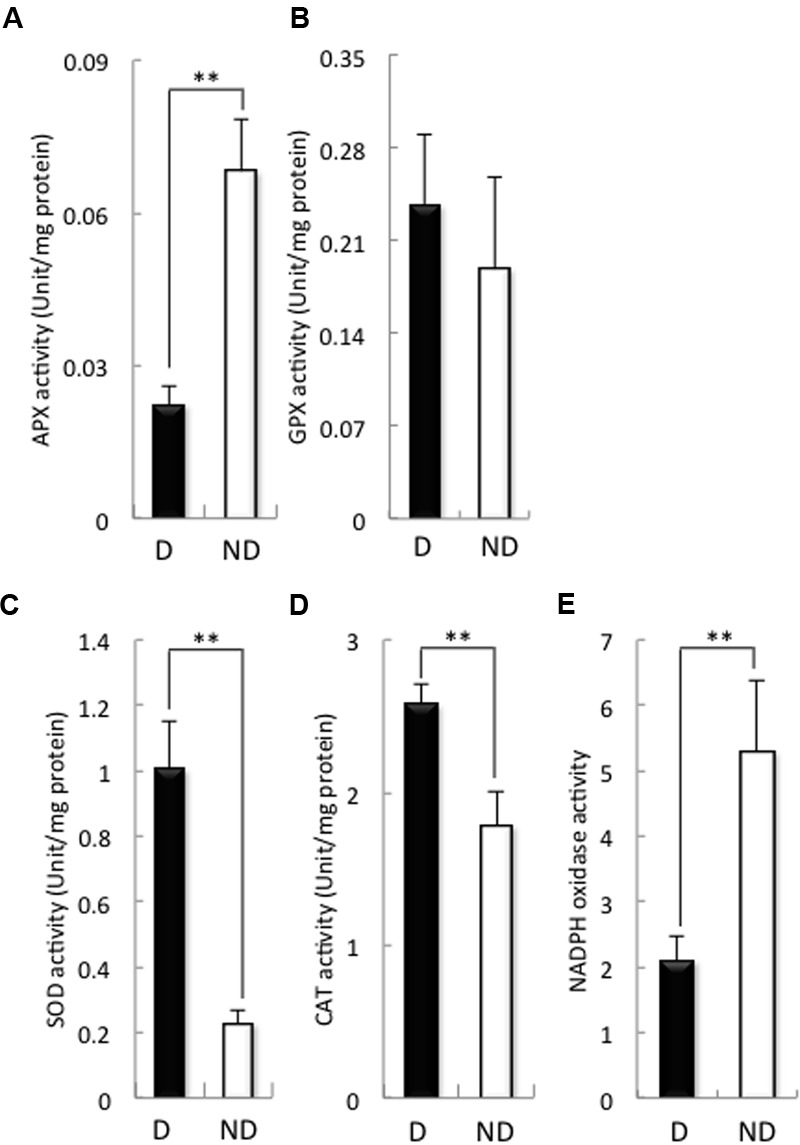
**Activity of antioxidant enzymes and NADPH oxidase in dormant and non-dormant seeds.** Embryos removed from seeds after 48 h imbibition were used. **(A)** APX; **(B)**, GPX; **(C)**, SOD; **(D)**, CAT; **(E)**, NADPH oxidase. D, dormant seed; ND, non-dormant seed. (^∗∗^*P* < 0.01, Student’s test, *n* = 5).

Then, we examined the activities of antioxidant enzymes such as APX, GPX, SOD, and CAT, as well as the activity of NADPH oxidase (**Figure 3**), because the H_2_O_2_ contents of the embryos during germination were higher in non-dormant seeds than in dormant seeds (**Figure 1**). APX activity in the embryos of dormant seeds was significantly higher than that of the embryos of non-dormant seeds (**Figure 3A**), although GPX activity was the same in the embryos of both non-dormant and dormant seeds (**Figure 3B**). In contrast, SOD and CAT activities in the embryos of dormant seeds were significantly lower than those in the embryos of non-dormant seeds (**Figures 3C,D**). We previously reported that NADPH oxidase plays a role in barley seed germination through the regulation of GA and ABA signaling (Ishibashi et al., 2015). Therefore, we examined NADPH oxidase activity in the embryos of dormant and non-dormant seeds (**Figure 3E**). NADPH oxidase activity was significantly higher in non-dormant seeds than in dormant seeds. These results suggest that the production of ROS in the embryos of barley seeds after imbibition is regulated by SOD, CAT, and NADPH oxidase.

### Transient Expression of CAT in Immature Embryos Decreases Their Ability to Germinate

**FIGURE 4:**
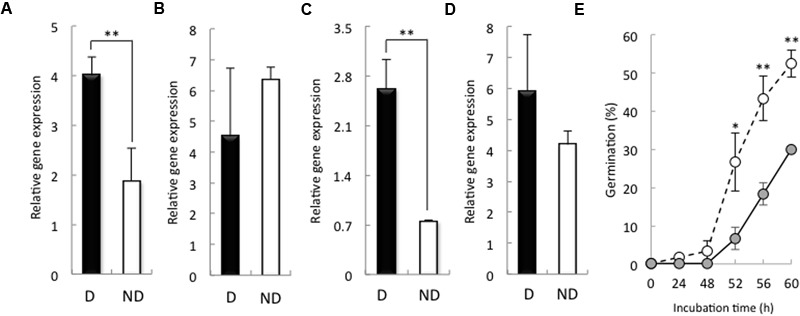
**Expression of HvSOD and HvCAT2 in dormant and non-dormant seeds and the germination percentage in the transient HvCAT2 expression assay.** Embryos removed from seeds after 24 h **(A,C)** and 48 h **(B,D)** imbibition were used to determine gene expression levels. **(A,B)** *HvSOD* expression; **(C,D)** *HvCAT2* expression. **(E)** Time course of the germination percentage after transformation with Ubi:HvCAT2 (closed circles) or Ubi:GUS (open circles). The barley cultivar Himalaya was used. D, dormant seed; ND, non-dormant seed. (^∗^*P* < 0.05, ^∗∗^*P* < 0.01, Student’s test, *n* = 5).

We examined the expression of *HvSOD* and *HvCAT* in the embryos of non-dormant and dormant seeds. After 24 h imbibition, the expression of *HvSOD* and *HvCAT2* was increased in the embryos of dormant seeds compared with that in the embryos of non-dormant seeds (**Figures 4A,C**), while, after 48 h imbibition, the expression of *HvSOD* and *HvCAT2* were high in the embryo of non-dormant and dormant seeds, respectively (**Figures 4B,D**). SOD catalyzes the reduction of the superoxide anion to hydrogen peroxide, and CAT catalyzes the decomposition of hydrogen peroxide to water. In wheat, the expression of CAT in dormant seeds was higher than that in non-dormant seeds during seed maturation (Ishibashi et al., 2008). In sunflower seed, relationship between CAT activity and germination is a quite close (Bailly et al., 1998). Additionally, Bahin et al. (2011) have reported that the CAT regulates hydrogen peroxide content in embryo of barley dormant seed. Therefore, we focus on the role of CAT in barley seed dormancy and germination. To examine the function of HvCAT2, which directly scavenges hydrogen peroxide, in barley seed germination, we transiently overexpressed HvCAT2 in immature embryos and examined their germination. HvCAT2 driven by the maize (*Zea mays*) ubiquitin promoter (Ubi:HvCAT2) was directly bombarded into immature embryos isolated from Himalaya seeds at approximately 10 days after anthesis. In general, cultured immature wheat embryos germinate precociously (Williamson et al., 1985). However, we observed a significant decrease in the germination percentage of immature embryos that were transformed with Ubi:HvCAT2 (**Figure 4E**). In contrast, immature embryos transformed with the control construct β-glucuronidase (GUS) driven by the maize ubiquitin promoter (Ubi:GUS) germinated well (**Figure 4E**). Nakamura et al. (2011) previously reported that the Ubi:TaMFT construct, in a similar transient assay, completely suppressed the germination of immature embryos of wheat because the TaMFT protein moved from the scutellum and coleorhiza to other parts of the seed. The partial effect of Ubi:HvCAT2 in our study was likely caused by scavenging H_2_O_2_ which across cell membranes in plants (Hooijmaijers et al., 2012), even though HvCAT2 cannot move from the scutellum and coleorhiza to other parts of the seed.

### ABA Regulates the Expression of *HvCAT2* through HvABI5

**FIGURE 5:**
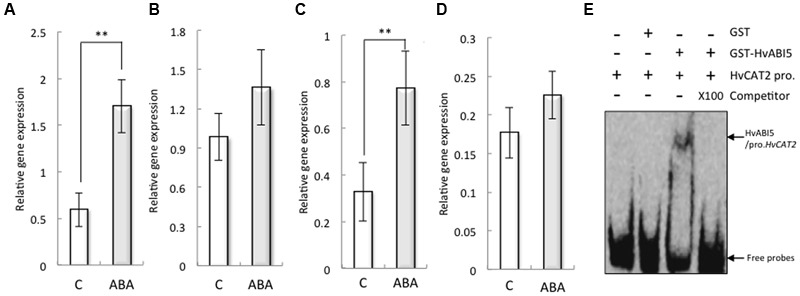
**Expression of HvCAT2 and HvABI5 in non-dormant seeds treated with 50 μM ABA and the direct interaction between HvABI5 and the HvHAT2 promoter as determined by EMSA.** Embryos removed from seeds after 24 h **(A,C)** and 48 h **(B,D)** imbibition were used to determine gene expression levels. **(A,B)** *HvCAT2;* **(C,D)** *HvABI5*. **(E)** The retarded DNA-protein complex was reduced by competition using either the wild-type or the unlabeled probes at a 100× molar excess. The arrows indicate the positions of the shifted bands and free probes, respectively. (^∗∗^*P* < 0.01, Student’s test, *n* = 5).

Abscisic acid significantly increased the expression of *HvCAT2* in the barley seed embryo (**Figures 5A,B**). To explore the underlying mechanisms by which ABA regulates the expression of *HvCAT2* during seed germination, we analyzed the promoter sequences of HvCAT2 and found an ABA response element (ABRE), which is a *cis*-acting element recognized by several bZIP transcription factors, such as ABI5, AREB1, AREB2, and ABF3, that function in ABA signal transduction (Supplementary Figure S5; Finkelstein and Lynch, 2000). These bZIP transcription factors mediate downstream gene expression in *Arabidopsis* upon binding to the ABRE (Furihata et al., 2006; Yoshida et al., 2010). Our finding suggests that the expression of *HvCAT2* may be regulated by bZIP transcription factors involved in ABA signaling. Indeed, the expression of *HvABI5* in the barley seed embryo was increased by ABA (**Figures 5C,D**). Therefore, to test this possibility, we used EMSA analyses to investigate whether HvABI5 directly binds to the promoter of HvCAT2 containing the ABRE element (**Figure 5E**). The recombinant protein of GST–HvABI5 showed binding activities to HvCAT2 promoter fragment, and the DNA-binding activity was suppressed by competitor.

## Discussion

The present study demonstrates that the balance between ABA and ROS in the barley seed embryo is involved in the regulation of seed dormancy and germination. The first line of evidence supporting this conclusion is the relationship between seed dormancy and H_2_O_2_ level, because the degree of seed dormancy was dependent on the H_2_O_2_ levels in the embryo after imbibition (**Figure 1** and Supplementary Figure S2). Additionally, germination rate of dormant seeds were promoted by H_2_O_2_ and suppressed by sodium ascorbate, while that of non-dormant seeds were suppressed by sodium ascorbate, but not promoted by H_2_O_2_ (**Figure 2A** and Supplementary Figure S3). There have been several reports about the relationship between ROS and seed dormancy and germination, many of which are focused on phytohormone signaling and ROS involvement (Oracz and Karpiński, 2016). H_2_O_2_ has been implicated in dormancy alleviation via its activation of GA signaling and synthesis rather than repression of ABA signaling (Bahin et al., 2011). In sunflower seed, however, ROS interact at the transcriptional level with the ABA signaling pathway (El-Maarouf-Bouteau et al., 2015). We previously reported that ROS produced by NADPH oxidase in the embryos of barley seeds promoted the catabolism of ABA (Ishibashi et al., 2015). In the present study, NADPH oxidase activity in the embryos of non-dormant seeds was higher than that in the embryos of dormant seeds (**Figure 3E**). In non-dormant seeds, ABA content in the embryos treated with ascorbate was higher than that in treated with distilled water, whereas, in dormant seeds, the ABA content in the embryos treated with H_2_O_2_ was lower than in treated with distilled water (**Figures 2C,D**). In addition, the regulation of ABA in the embryos of both dormant and non-dormant seeds could be attributed to the induction of *HvABA8*′*-OH1* by ROS (**Figures 2G,H**). Two genes encoding ABA8′-OH have been identified in barley, and *HvABA8*′*OH1* is predominantly expressed in the embryos of imbibing seeds (Millar et al., 2006). Of note, *HvABA8*′*OH1* expression is closely associated with ABA level and germination capacity (Chono et al., 2006). Consistent with these facts, the knocking down of *ABA8*′*OH1* leads to increased seed ABA levels and to enhanced dormancy (Gubler et al., 2008). ROS produced after imbibition is involved in the regulation of barley seed dormancy and germination through the catabolism of ABA; therefore, we focused on the mechanism of ROS production. It had been reported that ROS generated by NADPH oxidases regulate barley seed germination through GA/ABA metabolism and signaling in the embryo and aleurone cells (Ishibashi et al., 2015). In the present study, H_2_O_2_ content and NADPH oxidase activity in the embryos of dormant seeds were lower than those in the embryos of non-dormant seeds (**Figures 1B,C, 3E**). In the embryos of barley seeds, ABA suppressed the activity of NADPH oxidases (Ishibashi et al., 2015). The decrease in ABA content by catabolism in the embryo after imbibition is promoted by ROS production through NADPH oxidase. In *Arabidopsis*, ABA treatment induces the expression of *NADPH oxidases* (*AtrbohD* and *AtrbohF*) in guard cells (Kwak et al., 2003). The ROS enhanced by NADPH oxidase could result in enhanced ABA accumulation, whereas enhanced ABA could result in enhanced ROS production in guard cells, creating a positive feedback loop to mediate stomatal closure (Mittler and Blumwald, 2015). Our results indicate that in the embryo after imbibition, ROS and ABA act antagonistically to regulate seed dormancy and germination, although this suggestion is inconsistent with other reports.

In this study, we also examined the activity of the antioxidant enzymes SOD, CAT, and peroxidases (APX, GPX) that can scavenge ROS. The CAT activities in the embryos of the dormant seeds were significantly higher than those in the embryos of the non-dormant seeds (**Figure 3D**). These results suggest that the H_2_O_2_ contents in the embryos of dormant and non-dormant seeds depend on CAT activity. CAT is reported to play an important role in the seed germination process. CAT expression levels increase in germinating sunflower seeds prior to radicle protrusion, with a concomitant decrease in H_2_O_2_ content (Bailly, 2004). A similar increase in CAT activity has been described during the germination of maize, soybean, *Arabidopsis*, and sweet corn seeds (Puntarulo et al., 1991; Gallardo et al., 2001; Posmyk et al., 2001; Chiu et al., 2002). In dormant barley seeds, H_2_O_2_ treatment induced an increase in CAT activity that was associated with accumulation of the *HvCAT2* transcript in the embryos (Bahin et al., 2011). Barley has two CAT isozymes, HvCAT1 and HvCAT2; however, *HvCAT1* was not expressed in our embryos (data not shown). In the present study, the expression of *HvCAT2* in the embryos of dormant seeds was significantly higher than in those of non-dormant seeds (**Figure 4A**). In nine cultivars that were at different stages of dormancy, *HvCAT2* expression correlated with the germination index except for one cultivar (Supplementary Figure S6). In addition, precocious germination of isolated immature embryos was suppressed by the transient introduction of HvCAT2 driven by the maize (*Zea mays*) ubiquitin promoter (**Figure 4E**). These results indicated that CAT is involved in germination of barley seed after imbibition through the regulation of ROS.

The expression of *HvCAT2* was suppressed by ABA (**Figures 5A,B**, also Bahin et al., 2011); therefore, we investigated the promoter sequence of *HvCAT2*. The promoter sequence included an ABA-responsive element (ABRE), an ABRE-related coupling element (CE), and RY repeat motifs (Supplementary Figure S5). Interestingly, the promoter of *Sdr4*, which regulates rice seed dormancy, has seven RY repeats, which are important for seed-specific gene expression and are the target of the VP1/ABI3 subfamily of B3 domain transcription factors (Baumlein et al., 1992), along with the ABRE and CE (Sugimoto et al., 2010). The dormancy state is characterized by the transcription of genes with large numbers of ABRE sequences to which transcription factors like ABI5 bind to regulate seed dormancy (Finch-Savage and Leubner-Metzger, 2006). In the present study, the expression of HvABI5 in the embryos was increased by ABA (**Figure 5C**), but not HvVP1 (data not shown). Moreover, we determined that recombinant HvABI5 binds to the HvCAT2 promoter including the ABRE (**Figure 5E**). These results suggest that the expression of *HvCAT2* is regulated through HvABI5 induced by ABA.

**FIGURE 6:**
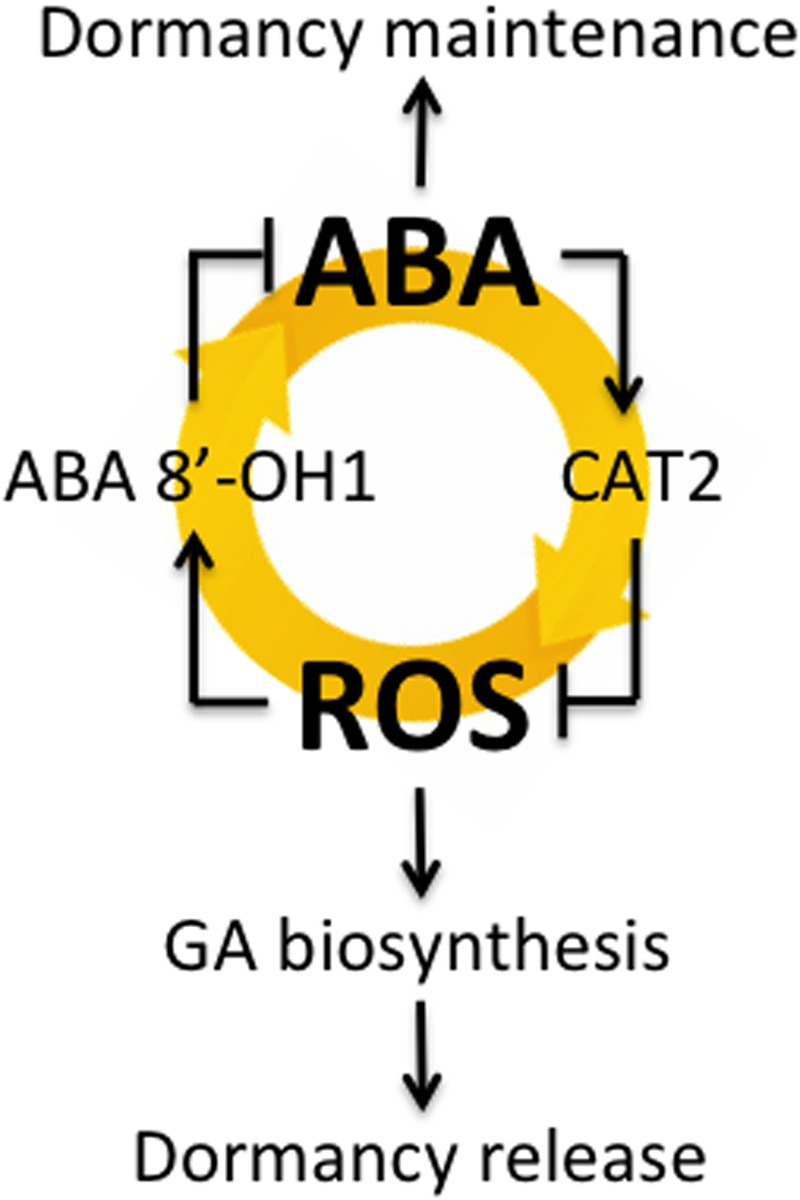
**The interrelationship between ABA and ROS involved in barley seed dormancy and germination**.

Bahin et al. (2011) suggested that H_2_O_2_ plays a role in the alleviation of barley seed dormancy through the activation of GA signaling and/or biosynthesis rather than through the inhibition of ABA signaling. They found that exogenously applied H_2_O_2_ does not influence ABA biosynthesis and signaling but does have a pronounced effect on GA signaling, resulting in the modulation of hormonal balance and in subsequent germination initiation. However, by using dormant and non-dormant seeds in this study, we found that H_2_O_2_ regulated ABA content through HvABA8′-OH1-mediated catabolism of ABA (**Figures 2C,D,G,H**). Liu et al. (2010) proposed a hypothetical model that explains the interrelationships between H_2_O_2_ and NO in the regulation of *Arabidopsis* seed germination in terms of the joint actions of ABA and GA. According to this model, H_2_O_2_ can interrupt the dormancy of *Arabidopsis* seeds through two pathways. The first pathway relies on the enhancement of ABA catabolism and GA biosynthesis. The signaling molecule (NO) does not regulate GA biosynthesis directly but instead acts as a temporary signaling molecule involved in the H_2_O_2_ regulation of ABA catabolism. The second pathway assumes negative regulation of GA biosynthesis by ABA. We previously reported that ROS induced by NADPH oxidase regulate barley seed germination by promoting ABA catabolism and GA biosynthesis (Ishibashi et al., 2015). Our results in this study suggest that dormant seeds maintain high ABA contents, promoting *HvCAT2* expression through ABI5 for H_2_O_2_ catabolism. In addition, ABA catabolism through HvABA8′OH1 was promoted by H_2_O_2_. Therefore, dormant seeds had low ROS contents with decreased *HvABA8*′*OH1* expression, which maintained high ABA contents. Taken together, our findings suggest that the interrelationship between ABA and ROS may play an important role in seed dormancy and germination, as shown in **Figure 6**, although it should also be added that the mechanism might be included in seedling stage.

## Author Contributions

Conceived and designed the experiments: YI, TY, and MI-I. Performed the experiments: YI, SK, MS, NA, KK, RT, and GW. Analyzed the data: YI, NA, TY, and MI-I. Contributed reagents/materials/analysis tools: YI, SK, MS, NA, KK, RT, and GW. Wrote the paper: YI and MI-I.

## Conflict of Interest Statement

The authors declare that the research was conducted in the absence of any commercial or financial relationships that could be construed as a potential conflict of interest.

## Abbreviations

ABA: abscisic acid
ABI5: ABA insensitive 5
APX: ascorbate peroxidase
CAT: catalase
EMSA: electrophoretic mobility shift assay
GA: gibberellin
GPX: glutathione peroxidase
H_2_O_2_: hydrogen peroxide
PHS: pre-harvest sprouting
ROS: reactive oxygen species
SOD: superoxide dismutase
