# Supplementary material for: The Interrelationship between Abscisic Acid and Reactive Oxygen Species Plays a Key Role in Barley Seed Dormancy and Germination
Source: Front Plant Sci. 2017 Mar 21;8:275. doi: 10.3389/fpls.2017.00275 (PMC5359625; doi:10.3389/fpls.2017.00275)
Supplement: Supplementary file 1 [file Table_1.PDF]

Supplementary Table S1. List of primers and probes used in this research.

**Genes** (Accessions)	**Sequences**
Hvactin (AY145451)	F: GCCGTGCTTTCCCTCTATG
	R: GCTTCTCCTTGATGTCCCTTA
HvCAT2 (U20778)	F: CCATAAGGGACCATGCATGCCAGCTAC
	R: GCTCGAAGCACCCACTTTAGTTTAAGC
HvABI5 (AY150676)	F: GTGCTTACGAAGAACGCTGACCCG
	R: CCTCGACTACACACTAGCCTACAGGTC
HvVP1 (AJ431703)	F: GACCTGCCGCGGTTCTTCATGGAGTGG
	R: GCCTCCTGATCCATGGCGCCGAC
HvSOD (AK252295)	F: CCGAAGATGAAATCCGCCAT
	R: CGGCCAATGATTGAATGTGG
HvNCED1 (AB239297)	F: CCAGCACTAATCGATTCC
	R: GAGAGTGGTGATGAGTAA
HvABA8'OH1 (AK333121)	F: AGCACGGACCGTCAAAGTC
	R: TGAGAATGCCTACGTAGTG
gus (AB489142)	F: CATGAAGATGCGGACTTAGC
	R: ATCCACGCCGTATTCGG
HvCAT2-Apa I	F: TATAGGGCCCATGGATCCCTGCAAGTTC
HvCAT2-Sac I	R: GATCGAGCTCTCACATGCTTGGCTTCAC
HvABI5-fusPro.	F:ATCTGGTTCCGCGTGGATCCATGGACTTCAGGAGCAGCAACG
	R:ATGCGGCCGCTCGAGTCGACTCACCAGGGCCCGGTCAG
HvCAT2-probe	F:CACACACACGCACTACGTTCCGAGCATTGCGTCGCGGTGGGTGAG
	R:CTCACCCACCGCGACGCAATGCTCGGAACGTAGTGCGTGTGTGTG
