# Supplementary material for: The Interrelationship between Abscisic Acid and Reactive Oxygen Species Plays a Key Role in Barley Seed Dormancy and Germination
Source: Front Plant Sci. 2017 Mar 21;8:275. doi: 10.3389/fpls.2017.00275 (PMC5359625; doi:10.3389/fpls.2017.00275)
Supplement: Supplementary file 2 [file Presentation_1.PDF]

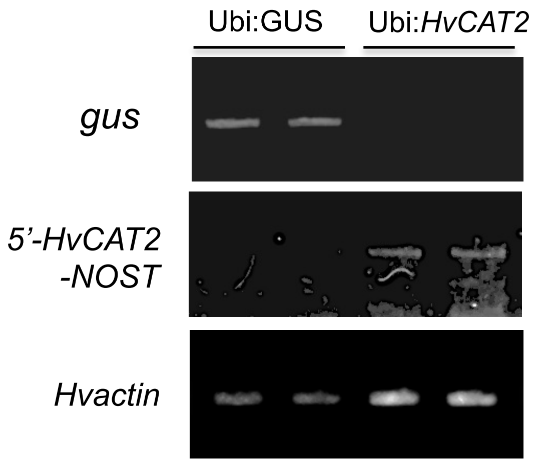

Supplementary Figure S1. RT-PCR in immature embryos transformed by particle bombardment.

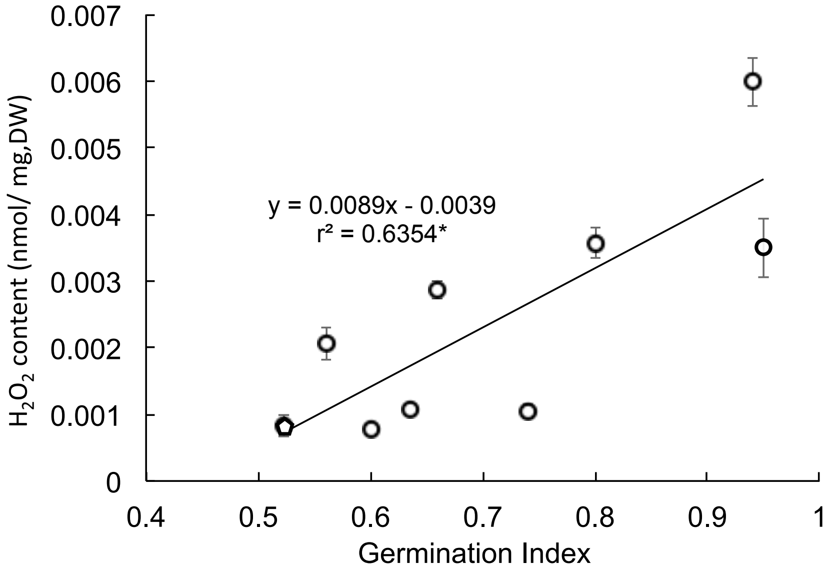

Supplementary Figure S2. Correlation between germination index and hydrogen peroxide content in embryo of nine barley cultivars. Hydrogen peroxide contents in embryo after 24 h imbibition were measured. Minorimugi, ELISE, Haruna906, Himalaya, Seijo17, Amaginijo, Kantonijo25, Ichibanboshi and Chikukei9713 were used. (**P* <0.05, *n* = 5).

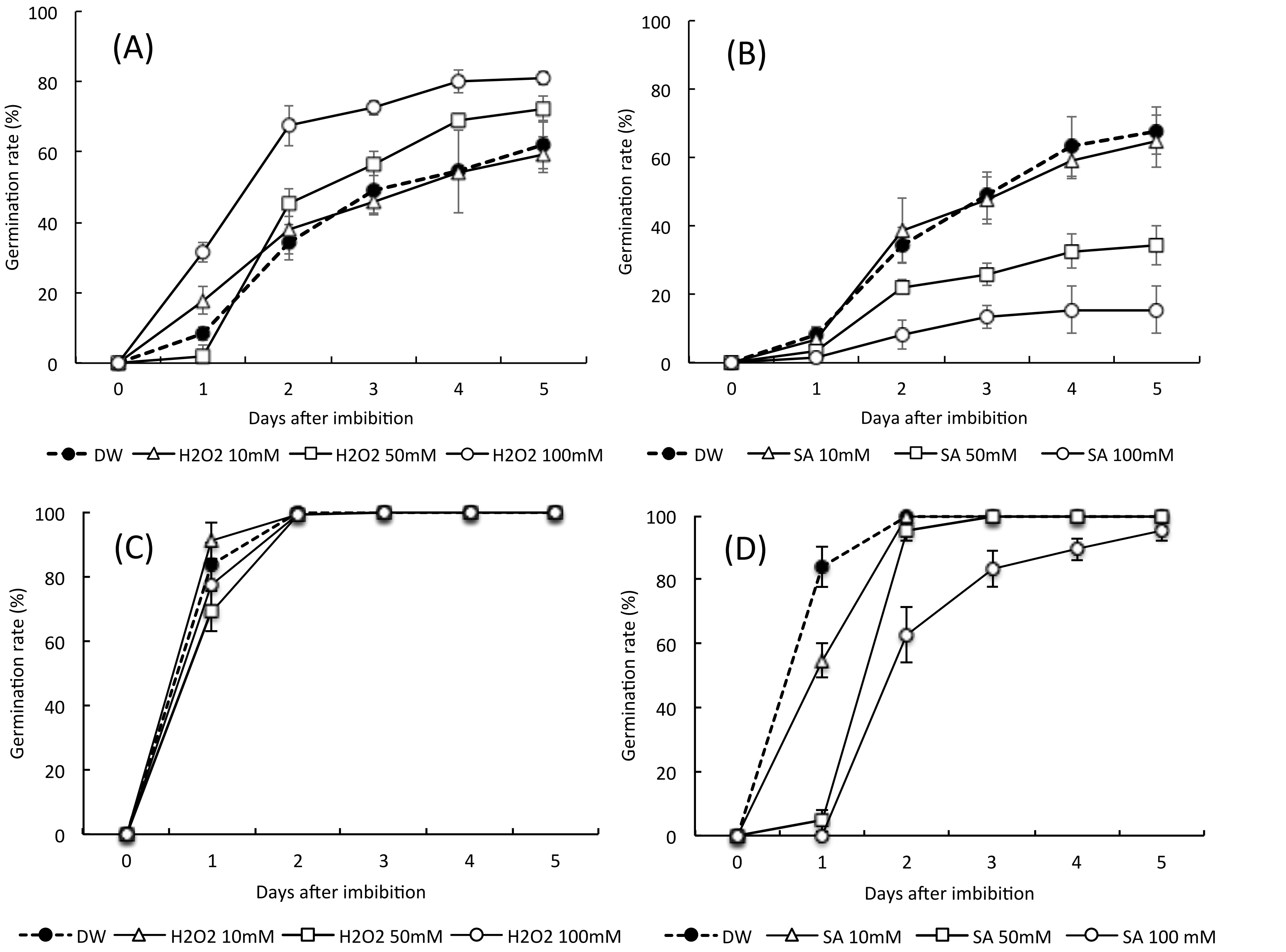

Supplementary Figure S3. Germination rate of barley (cv. Himalaya) dormant seed (A, B) harvested in 2016 and non-dormant seed (C, D) harvested in 2013 treated with (A, C) hydrogen peroxide (H2O2) and (B, D) sodium ascorbate (SA).

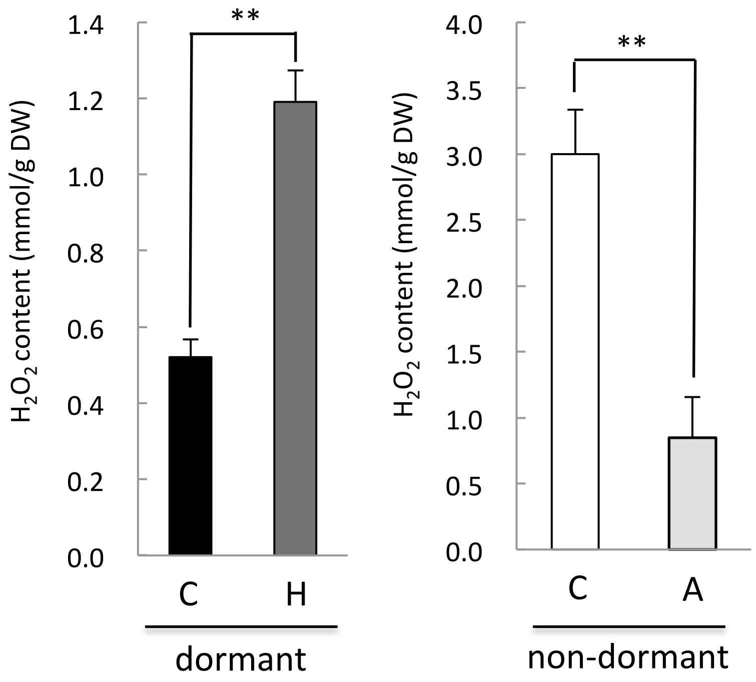

Supplementary Figure S4. Hydrogen peroxide contents in dormant and non-dormant seeds. Embryos removed from seeds after 48 h imbibition were used to determine hydrogen peroxide contents . C, control (distilled water); H, 100 mM hydrogen peroxide; A, 20 mM sodium ascorbate. (***P* < 0.01, Student's test, *n* = 5).

ATAATTATGTTGGGAAAAACAAATCTAGTTCTTTCCAACTATATATA
TTTAGGTACAGAAAAATTCTCAGCTGAAAAATAAATTAACATGTAA
GGACACCTCGGATAGACAAGCTTATCTCTTTTTTTTCCAGTGAGCTA
GACAAGATCATCATTATCCATC**CCACC**GTTGATCACCGATTTTTACG
CACACAAGTCGCGATTTTTACACACACACGCACT**ACGTTC**CGAGCA
TT**GCGT**CGCGGTGGGTGAGTGCAACAGATCATTGTCCAGAAGGAG
ACA**CATGCA**CTGATGCACGCATAGCAGCCTCTTTTGTATTTGCACG
GAACCAGATGACGATGGGGCGACGATCGGTCGATGCGGTGCACCC
GTCCCAGCTACAGACTTCTCCAC**ACGTGG**CGGGC**CCACC**ACACCT
TATCCTCTCTTGTCTC**CATGCA**GGCCCACCCACCTGTCAGAGGGTG
GTCACGGCAGCCGGAGCGGCACGCCACCTCACGCCCCCGCCGTTA
TTTAAGAAGAAGACCAGGATGGAGCTCCAAGCAGAGCAGA
TCAGTGCAGTGAGAGTGAGTGAGTGAGTGAGCGAGCGACA
CTCCCCACCCCCAGCTTAGCTACGCTACTTCACTCACTCGAG
GAAGG**ATG**

Supplementary Figure S5. Sequence of HvCAT2 promoter. Blue, red and purple sites show coupling elements (CE), ABA responsive elements (ABRE) and RY repeat (RY), respectively. These cis-elements were investigated using PlantCARE (http://bioinformatics.psb.ugent.be/webtools/plantcare/html/)

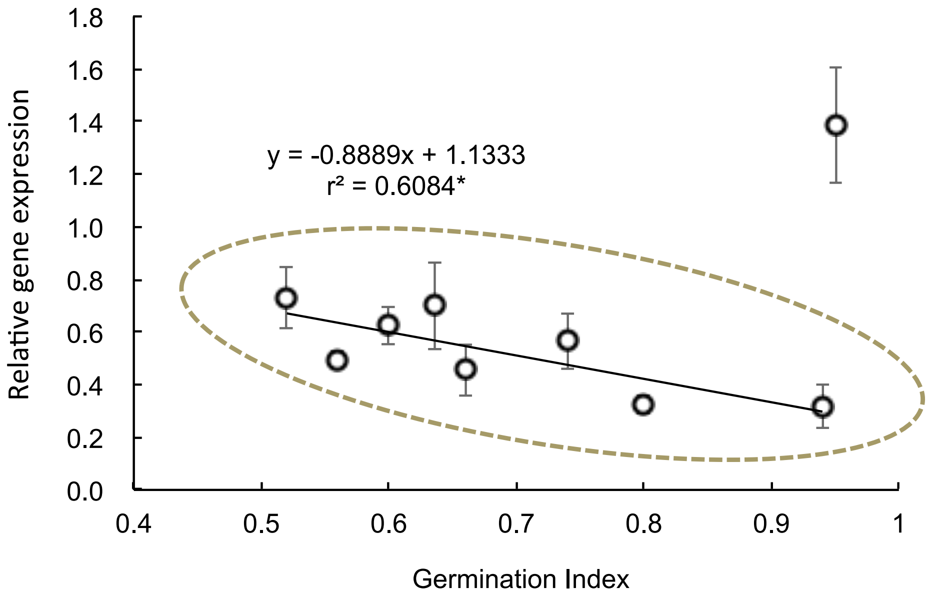

Supplementary Figure S6. Correlation between germination index and expression of *HvCAT2* in embryo of nine barley cultivars. The expression of *HvCAT2* in embryo after 24 h imbibition were measured. Minorimugi, ELISE, Haruna906, Himalaya, Seijo17, Amaginijo, Kantonijo25, Ichibanboshi and Chikukei9713 were used. (*P <0.05).
